# Past President's Address

**DOI:** 10.4103/0019-5049.60489

**Published:** 2010

**Authors:** Manjushree Ray

**Affiliations:** President, ISA- 2009 E mail: manjushriray@hotmail.com

Respected chief guest, guest of honour, dignitaries on and off the dais, invitees, former presidents, governing council members, fellow members of Indian Society of anaesthesiologists (ISA), members of industry, members of media, ladies and gentleman, on behalf of the organising committee, I extend a warm welcome to you all on the occasion of the 57^th^ annual conference of ISA.

Today, standing on this podium, the year that passed runs through my mind, before my eyes. Every year, the past presidents have stood under this prestigious spotlight, created visions and inched their way towards a brighter future. I hope to have achieved my minute role in this effort to make the world a better place to live in.

This ISA was founded way back on December 30, 1947, by Dr. G S Ambardekar, Dr. B.N. Sirkar and 12 other visionaries. First, I bow my head in respect before them. Our rich history remains the most important and integral part of our present and future. In 1949, the first conference was held in Bombay with ASI and by 1965 the first independent conference was held in Hyderabad. The Indian Journal of Anaesthesia was published in 1953 with Dr. M.C. Ganguly as its Editor.

Gradually our society became rich with bimonthly publications of the journal, an annual conference with a pre-conference Continuing Medical Education (CME) and guidelines and protocols for the conference to avert frightful mistakes of the past.

With the turn of the millennium, till date, which we proudly call the watershed in the history of ISA, we worked on all fronts. The ISA WFSA-sponsored CME programmes were started with the intention of providing updated knowledge to all anaesthesiologists, especially those who are working in the rural set up. The PG education received special attention and our long term dream has started taking its first step in the form of Indian College of Anaesthesia – an Academic Wing of ISA.

I proudly announce that 160 new postgraduate seats have been created in different Universities this year. This has only been possible because of your initiatives and persuasion. I must also inform you that the new MCI guidelines that one professor can be a guide to two postgraduate students every year will definitely help us achieve our mission – the World Health Organisation (WHO) recommendation of one anaesthesiologist every 30,000 – 35,000 population.

The ISA has, in the past year, with the help of renowned teachers and academicians, tried to establish a uniform postgraduate curriculum and standard throughout the country.

On November 21, 2008, the Indian College of Anaesthesiologists was registered. In our last General Body meeting, a few senior members had suggested some of rules, regulations and byelaws and these modifications in the laws of the ICA have been accepted by the ICA body. I sincerely thank everybody and eagerly wait to see the ICA flourish into a hub of academic activities from a dream, through a vision into a reality.

Indian anaesthesiologists have so long been following western guidelines and protocols for perioperative management of patients. India has different ethnic and anthropological variations, which distinctly affect the perioperative outcome. We have been aware of these variations and have acted accordingly for years in the practical and clinical settings. Today we are gathering all our experiences and finally putting forth our very own guidelines and protocols designed to cater to the needs of our very own people. A booklet related to the guidelines and protocols was published on the foundation day of ICA. The CPR guidelines have reached a final shape and are nearly ready for publication. I thank all the eminent anaesthesiologists, from all over the country, who have taken pains to establish this change.

Unfortunately, another dream is still unrealized. There is very little original research in the field of anaesthesia in our country. From my meagre experience, I would rather admit that motivation, perseverance and aptitude help more than availability of resources. Even with limited funding, a lot of good original work can be carried out. Good work done by us will inspire our young budding anaesthesiologists, to indulge in this rewarding activity. Search for cost-effective treatment protocols will always remain a vast area of research in this part of the world. To improve scientific deliberations many awards were initiated to recognize the talent and stimulate young minds to conduct research.

The year 2007–2008 has been earmarked by WHO as safe surgery, saves lives. Safe anaesthesia practice is an integral part of safe surgical practice. As a surgical team member, we the anaesthesiologist have immense responsibility to attain the WHO objectives. WHO has given maximum stress on safe anaesthesia practice, clearly mentioned that the anaesthesia team should use methods known to prevent harm from administration of anaesthetics while protecting the patient from surgical pain. Airway maintenance and management of perioperative blood loss are of paramount importance.

The World Federation of Societies of Anaesthesiologists (WFSA) has also been recommending International standard for a safe practice of Anaesthesia since long. This was again revised at the 2008 World Congress. In the last one year, in all the zonal and state conferences held in the different parts of our country, this has been one of our primary goals – to practice safe Anaesthesia. ISA strongly recommends implementation of safety check list, colour coding of drugs, syringe labelling, machine checks and avoiding complications in patients with co-morbid condition. Small work goes a long, long way, and nothing comes before life.

In this wake, I must mention the latest legal developments regarding the short term Anaesthesia course. Honourable High Court of Delhi, after hearing arguments from all concerned on conduct of short term training in Anaesthesia by Government of India, proclaimed that training for life saving anaesthesia skill, emergency obstetric care will continue till the requirement is fulfilled. However, so trained personnel will be permitted to give anaesthesia only in government rural health centres. Any deviation in working outside the specified area or in any other surgical procedure may lead to penal disciplinary action. All ISA members are therefore requested to remain vigil and if any incident is witnessed, kindly notify the head office immediately.

Now, to admit the work that has still remained unfulfilled regarding our society - the ISA website has been formed; a regular publication of newsletter has also been started to communicate better with all members. However, our goal to create a paperless office by using the e-facilities is still far from achieved. I request all members to help the office-bearers consolidate their efforts.

All of you are aware that the ISA has been divided into six zones to smoothen its activities. Last year we had zonal meetings and coordinators were elected from East Zone, Central Zone and South Zone. We are still waiting for response from the North East, West and North Zone. The moment information from all the zones reaches us; we will start elections of governing council members at the zonal level so that a proper representation as per the membership strength of the different zones may be made.

No academic organisation can remain an island. Public service activities should form an integral part of our work schedule. Efforts have been made by the state and city branches to educate the common and non-academic people about CPR and primary care of trauma victims. We need to develop public awareness campaigns and educate the society. More issues that directly affect the health of the population should also be taken up as a cause

But, well, charity should begin from home. The Family Benevolent Fund has been started for the benefit of our members and their families. It is functioning well all over the country. I request all members to join it and utilize it properly.

Looking back, a year ago, when I stood on this podium I wondered at the momentous responsibility that I shouldered. What I achieved in so short a time is very little. ICA is now a wing of the ISA. I have strived to pass on the message to all parts of India – Practice Safe Anaesthesia.

I look forward to handing over the torch, lit in 1947, to the next torch-bearer. I shall continue to render my humble services to the society whenever the need arises. Before concluding, I thank all members, past presidents, governing council members for all the love, help and cooperation that they have given me. So let us all dream big together and strive ahead to reach the rainbow.

Jai Hind

Long live ISA



